# Supplementary material for: Predictive Value of Reactogenicity for Anti-SARS-CoV-2 Antibody Response in mRNA-1273 Recipients: A Multicenter Prospective Cohort Study
Source: Vaccines (Basel). 2023 Jan 3;11(1):120. doi: 10.3390/vaccines11010120 (PMC9862064; doi:10.3390/vaccines11010120)
Supplement: Supplementary file 1 [file vaccines-11-00120-s001.zip › vaccines-2065304-supplementary.pdf]

**Supplementary Table 1.** Relationship between adverse events experienced after either dose (first or second dose) and immune response eight weeks after the first dose (approximately four weeks after the second dose; T2)

| Type of AE | SARS-CoV-2 antibody assay | subjects with AEs after 1st or 2nd dose | | *p*-value |
|---|---|---|---|---|
| | | no | yes | |
| Any local AE | Anti-S IgG (U/mL) | N=1<br>2618.18 | N=170<br>4422.83 (4094.49- 4778.59) | 0.307 |
| | $ND_{50}$ | N=0 | N=100 | NA |
| Injection site pain | Anti-S IgG (U/mL) | N=3<br>3820.32 (1688.88- 8643.71) | N=168<br>4420.79 (4087.90- 4779.69) | 0.625 |
| | $ND_{50}$ | N=1<br>3090.30 | N=99<br>2849.71 (2476.28- 3278.67) | 0.909 |
| Injection site redness/ swelling | Anti-S IgG (U/mL) | N=121<br>4302.29 (3938.22- 4698.94) | N=49<br>4744.60 (4049.49- 5560.32) | 0.257 |
| | $ND_{50}$ | N=64<br>3034.59 (2532.79- 3635.80) | N=35<br>2598.36 (2078.35- 3250.87) | 0.293 |
| Injection site motion limitation | Anti-S IgG (U/mL) | N=7<br>3802.77 (2542.14- 5687.22) | N=164<br>4437.11 (4099.21- 4802.86) | 0.435 |
| | $ND_{50}$ | N=0 | N=100 | NA |

| | | | | |
|---|---|---|---|---|
| Any systemic AE | Anti-S IgG (U/mL) | N=3<br>3894.04 (1630.80- 9300.37) | N=168<br>4418.76 (4086.96- 4778.59) | 0.672 |
| | $ND_{50}$ | N=0 | N=100 | NA |
| Fever (≥37.5°C) | Anti-S IgG (U/mL) | N=44<br>3944.57 (3332.73- 4668.74) | N=127<br>4582.47 (4204.36- 4994.59) | 0.093 |
| | $ND_{50}$ | N=21<br>2828.13 (2082.57- 3839.72) | N=79<br>2858.25 (2437.25- 3351.97) | 0.951 |
| Chill | Anti-S IgG (U/mL) | N=47<br>4030.88 (3419.01- 4753.35) | N=124<br>4561.42 (4183.12- 4975.08) | 0.158 |
| | $ND_{50}$ | N=23<br>2681.02 (2071.57- 3469.76) | N=77<br>2904.69 (2460.93- 3429.26) | 0.632 |
| Headache | Anti-S IgG (U/mL) | N=42<br>3939.13 (3292.30- 4713.03) | N=129<br>4574.04 (4204.36- 4976.22) | 0.099 |
| | $ND_{50}$ | N=21<br>2891.35 (2021.16- 4136.19) | N=79<br>2841.19 (2439.50- 3309.02) | 0.92 |
| Muscle pain | Anti-S IgG (U/mL) | N=12<br>4441.20 (3550.59- 5555.20) | N=159<br>4406.56 (4061.63- 4781.89) | 0.96 |
| | $ND_{50}$ | N=5<br>2727.09 (2054.94- 3619.93) | N=95<br>2858.25 (2413.79- 3308.26) | 0.717 |

| | | | | |
|---|---|---|---|---|
| Fatigue | Anti-S IgG (U/mL) | N=31<br>4487.45 (3657.63- 5506.81) | N=140<br>4392.38 (4039.24- 4776.39) | 0.833 |
| | $ND_{50}$ | N=16<br>2753.59 (1845.02- 4109.60) | N=84<br>2870.78 (2469.45- 3338.11) | 0.829 |
| Joint pain | Anti-S IgG (U/mL) | N=120<br>4350.11 (3954.58- 4788.30) | N=51<br>4550.93 (3989.33- 5191.59) | 0.599 |
| | $ND_{50}$ | N=69<br>2732.75 (2308.87- 3234.45) | N=31<br>3135.45 (2427.17- 4050.42) | 0.367 |
| Vomiting | Anti-S IgG (U/mL) | N=153<br>4470.95 (4114.34- 4859.59) | N=18<br>3917.42 (3212.18- 4777.49) | 0.300 |
| | $ND_{50}$ | N=89<br>2852.33 (2464.34- 3301.41) | N=11<br>2847.74 (1686.55- 4808.39) | 0.994 |
| Rash | Anti-S IgG (U/mL) | N=150<br>4420.79 (4082.25- 4786.30) | N=21<br>4330.12 (3244.890 5778.30) | 0.862 |
| | $ND_{50}$ | N=84<br>2930.89 (2505.53- 3428.47) | N=16<br>2470.02 (1831.05- 3331.96) | 0.373 |
| | | 2875.41 (2480.85- 3331.96) | 2669.93 (1639.08- 4349.11) | |

Data is presented as geometric mean titers (95% confidence interval)

AE, adverse event; NA, not available; $ND_{50}$, median neutralizing titer

**Supplementary Table 2.** Univariate and multivariate analyses for predictive factors of strong antibody response at each time point

| | Anti-S IgG at T1 | | *p*-value | Adjusted OR (95% CI) | Anti-S IgG at T2 | | *p*-value | Adjusted OR (95% CI) |
|---|---|---|---|---|---|---|---|---|
| | <250 U/mL (n=134) | ≥250 U/mL (n=43) | | | <5400 U/mL (n=111) | ≥5400 U/mL (n=60) | | |
| Age, year (SD) | 25.2 (3.6) | 25.9 (4.5) | 0.322 | 1.036 (0.953-1.127)[a] | 25.3 (2.7) | 25.5 (5.4) | 0.759 | 1.009 (0.931-1.094)[a] |
| | | | | | | | | 1.023 (0.944-1.110)[b] |
| Male | 39 (29.1%) | 14 (32.6%) | 0.667 | 1.223 (0.522-2.867)[a] | 72 (64.9%) | 46 (76.7%) | 0.111 | 1.525 (0.674-3.449)[a] |
| | | | | | | | | 1.716 (0.718-4.102)[b] |
| BMI, $kg/m^2$ (SD) | 21.3 (2.8) | 22.1 (3.0) | 0.103 | 1.108 (0.970-1.266)[a] | 21.8 (2.9) | 21.2 (2.9) | 0.212 | 0.969 (0.854-1.100)[a] |
| | | | | | | | | 0.982 (0.863-1.116)[b] |
| AE after dose 1 | | | | | | | | |
| Any AE | 134 (100%) | 42 (97.7%) | 0.243 | | 110 (99.1%) | 60 (100%) | 0.461 | |
| Any local AE | 132 (98.5%) | 42 (97.7%) | 0.568 | | 108 (97.3%) | 60 (100%) | 0.271 | |
| pain | 130 (97%) | 40 (93%) | 0.226 | 0.471 (0.097-2.288)[a] | 105 (94.6%) | 59 (98.3%) | 0.227 | 2.638 (0.297-23.399)[a] |
| swelling | 23 (17.2%) | 4 (9.3%) | 0.212 | | 18 (16.2%) | 9 (15.0%) | 0.835 | |
| Motion limitation | 119 (88.8%) | 39 (90.7%) | 0.490 | | 95 (85.6%) | 57 (95.0%) | 0.062 | |
| Any systemic AE | 118 (88.1%) | 37 (86.0%) | 0.728 | | 96 (86.5%) | 53 (88.3%) | 0.731 | |
| fever | 28 (20.9%) | 10 (23.3%) | 0.743 | | 19 (17.1%) | 16 (26.7%) | 0.140 | |
| chill | 30 (22.4%) | 7 (16.3%) | 0.391 | | 23 (20.7%) | 11 (18.3%) | 0.709 | |
| headache | 40 (29.9%) | 16 (37.2%) | 0.367 | | 32 (28.8%) | 22 (36.7%) | 0.293 | |
| Muscle pain | 98 (73.1%) | 32 (74.4%) | 0.868 | | 77 (69.4%) | 48 (80.0%) | 0.135 | |
| Fatigue | 78 (58.2%) | 26 (60.5%) | 0.794 | | 66 (59.5%) | 33 (55.0%) | 0.573 | |
| arthralgia | 13 (9.7%) | 4 (9.3%) | 0.602 | | 10 (9.0%) | 6 (10.0%) | 0.832 | |

| | | | | | | | | |
|---|---|---|---|---|---|---|---|---|
| vomiting | 3 (2.2%) | 0 (0.0%) | 0.432 | | 3 (2.7%) | 0 (0.0%) | 0.271 | |
| Rash | 10 (7.5%) | 3 (7.0%) | 0.609 | | 10 (9.0%) | 3 (5.0%) | 0.267 | |
| dyspnea | 4 (3.0%) | 1 (2.3%) | 0.648 | | 4 (3.6%) | 1 (1.7%) | 0.424 | |
| flushing | 4 (3.0%) | 1 (2.3%) | 0.648 | | 4 (3.6%) | 1(1.7%) | 0.424 | |
| Facial palsy | 4 (3.0%) | 1 (2.3%) | 0.648 | | 4 (3.6%) | 1(1.7%) | 0.424 | |
| paresthesia | 14 (10.4%) | 1 (2.3%) | 0.080 | | 12 (10.8%) | 3 (5.0%) | 0.200 | |
| Antipyretic use | 81 (60.4%) | 26 (60.5%) | 0.998 | 1.009 (0.491-2.073)[a] | 58 (52.3%) | 43 (71.7%) | 0.014 | 2.202 (1.110-4.367)[a] |
| AE after dose 2 | | | | | | | | |
| Any AE | | | | | 108 (97.3%) | 60 (100%) | 0.271 | |
| Any local AE | | | | | 107 (96.4%) | 57 (95.0%) | 0.470 | |
| pain | | | | | 104 (93.7%) | 57 (95.0%) | 0.510 | 0.511 (0.098-2.652)[b] |
| swelling | | | | | 21 (18.9%) | 17 (28.3%) | 0.158 | |
| Motion limitation | | | | | 98 (88.3%) | 54 (90.0%) | 0.734 | |
| Any systemic AE | | | | | 104 (93.7%) | 59 (98.3%) | 0.161 | |
| fever | | | | | 76 (68.5%) | 49 (81.7%) | 0.063 | |
| chill | | | | | 73 (65.8%) | 48 (80.0%) | 0.051 | |
| headache | | | | | 76 (68.5%) | 44 (73.3%) | 0.507 | |
| Muscle pain | | | | | 89 (80.2%) | 52 (86.7%) | 0.287 | |
| Fatigue | | | | | 86 (77.5%) | 48 (80.0%) | 0.702 | |
| arthralgia | | | | | 29 (26.1%) | 16 (26.7%) | 0.939 | |
| vomiting | | | | | 12 (10.8%) | 4 (6.7%) | 0.375 | |
| Rash | | | | | 6 (5.4%) | 5 (8.3%) | 0.330 | |
| dyspnea | | | | | 6 (5.4%) | 0 (0.0%) | 0.071 | |
| flushing | | | | | 1 (0.9%) | 0 (0.0%) | 0.649 | |

| | | | | |
|---|---|---|---|---|
| Facial palsy | 1 (0.9%) | 0 (0.0%) | 0.649 | |
| paresthesia | 5 (4.5%) | 3 (5.0%) | 0.578 | |
| Antipyretic use | 96 (86.5%) | 59 (98.3%) | 0.011 | 10.033 (1.185-84.924)[b] |

OR, odds ratio; SD, standard deviation; BMI, body mass index; AE, adverse event.

[a] Adjusted for age, sex, BMI, injection site pain after dose 1, and antipyretic use after dose 1

[b] Adjusted for age, sex, BMI, injection site pain after dose 2, and antipyretic use after dose 2

**Supplementary Figure 1.** Solicited adverse events after each dose

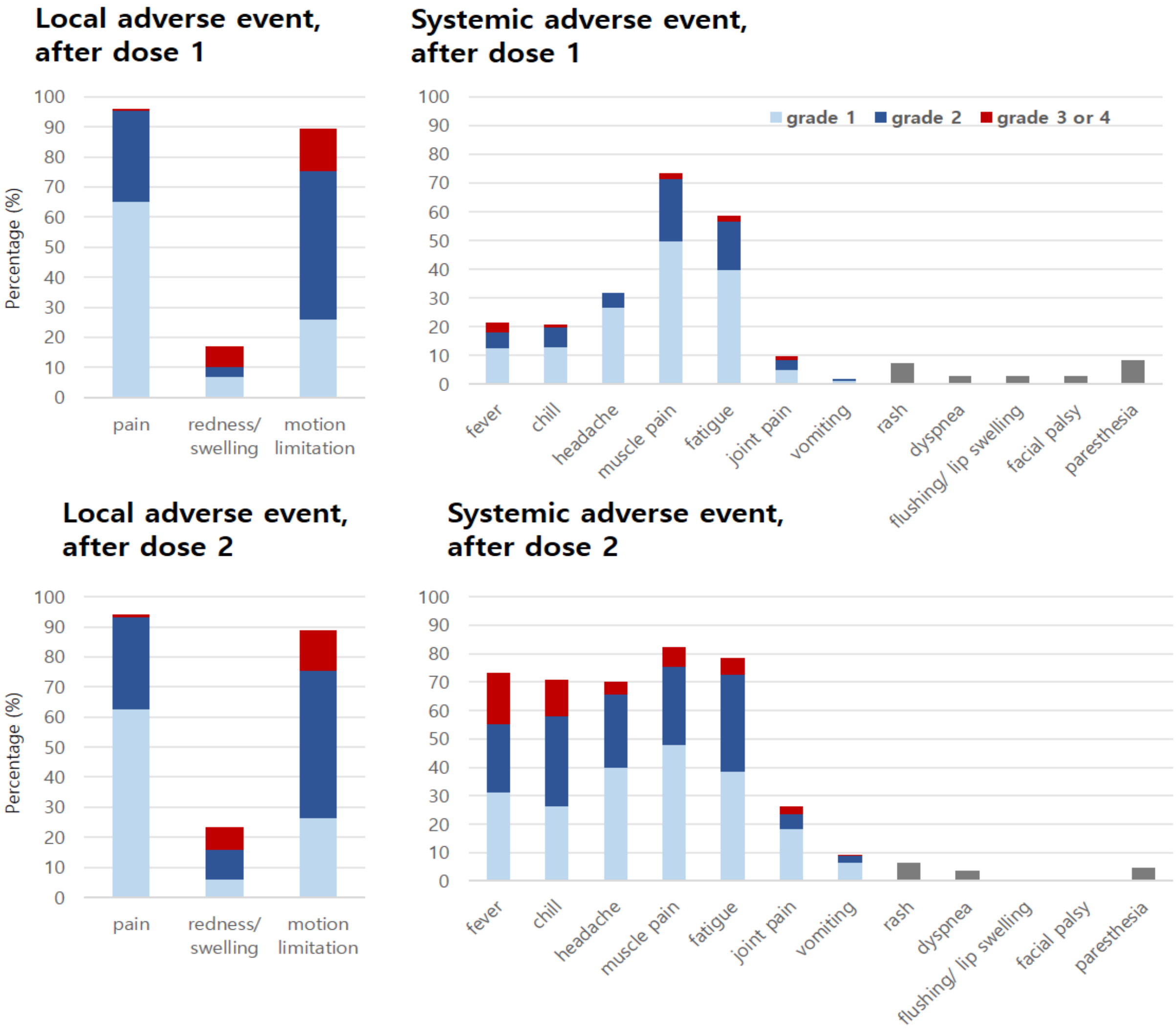

**Supplementary Figure 2.** Correlation between anti-SARS-CoV-2 S IgG and neutralizing antibody titer at each time point: (A) Four weeks and (B) Eight weeks after first injection.

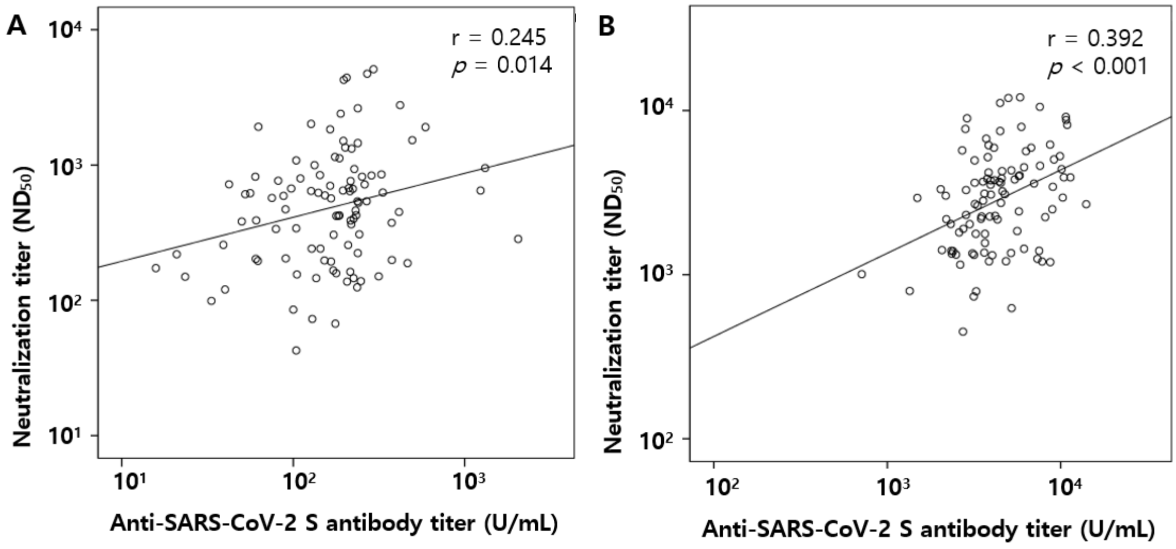

**Supplementary Figure 3.** Relationship of immune response with (A) highest severity of all adverse events, (B) highest severity of local adverse events, and (C) highest severity of systemic adverse events at each time point. Immune responses are presented as geometric mean titer.

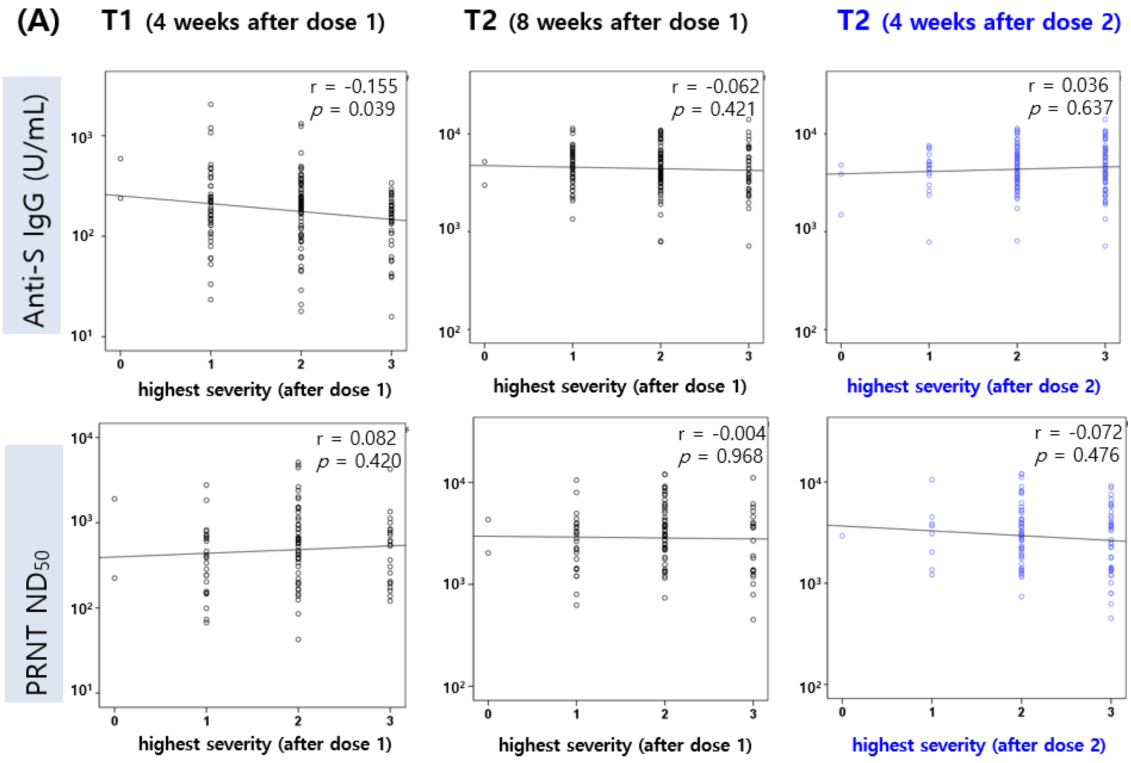

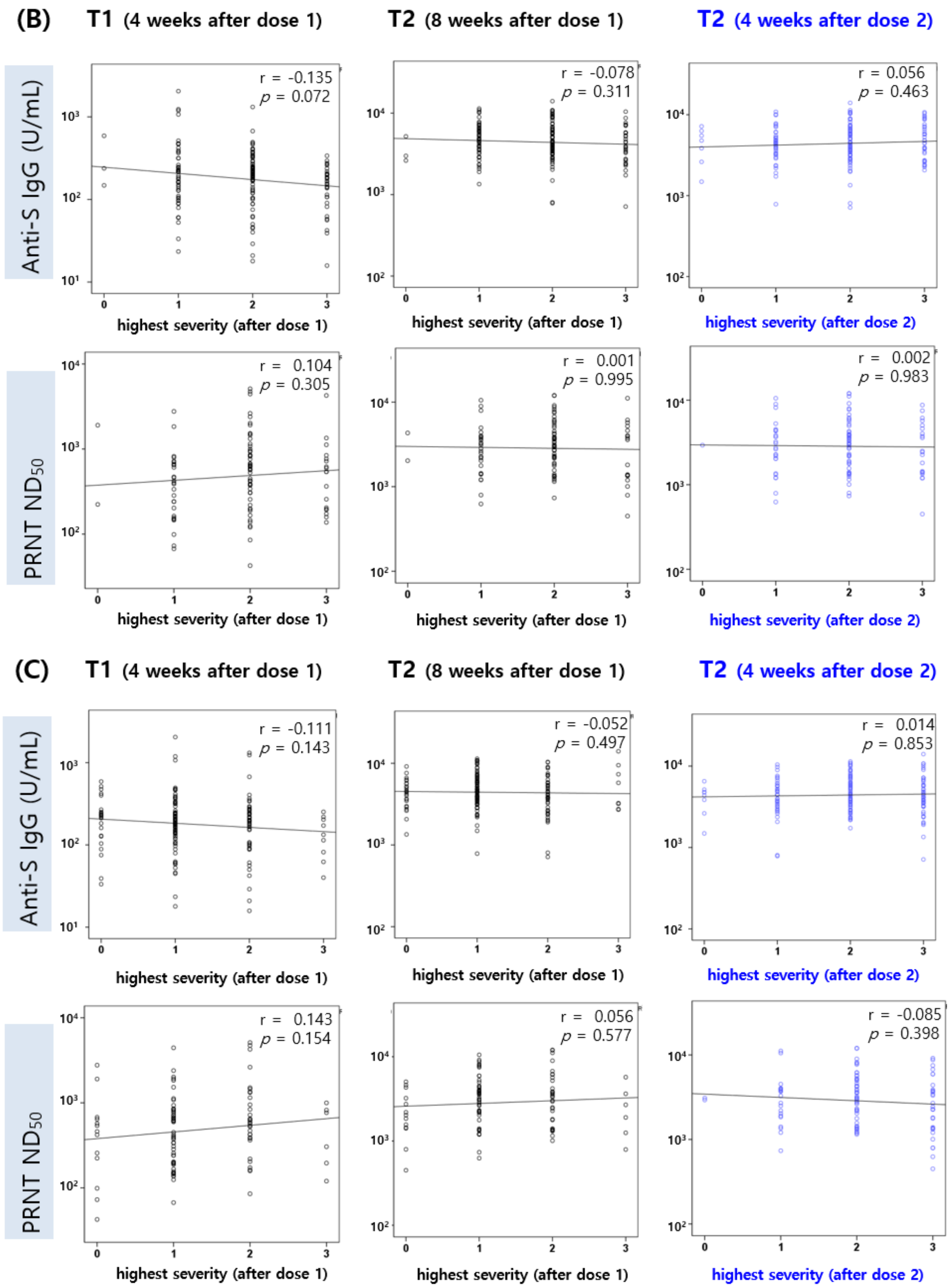
(B)
T1 (4 weeks after dose 1)
T2 (8 weeks after dose 1)
T2 (4 weeks after dose 2)
Anti-S IgG (U/mL)
PRNT ND50
r = -0.135
p = 0.072
r = -0.078
p = 0.311
r = 0.056
p = 0.463
r = 0.104
p = 0.305
r = 0.001
p = 0.995
r = 0.002
p = 0.983
highest severity (after dose 1)
highest severity (after dose 2)
(C)
T1 (4 weeks after dose 1)
T2 (8 weeks after dose 1)
T2 (4 weeks after dose 2)
Anti-S IgG (U/mL)
PRNT ND50
r = -0.111
p = 0.143
r = -0.052
p = 0.497
r = 0.014
p = 0.853
r = 0.143
p = 0.154
r = 0.056
p = 0.577
r = -0.085
p = 0.398
highest severity (after dose 1)
highest severity (after dose 2)

**Supplementary Figure 4.** Relationship of anti-SARS-CoV-2 S antibody response with (A) systemic SUM and SOM, and (B) localized SUM and SOM at each time point.

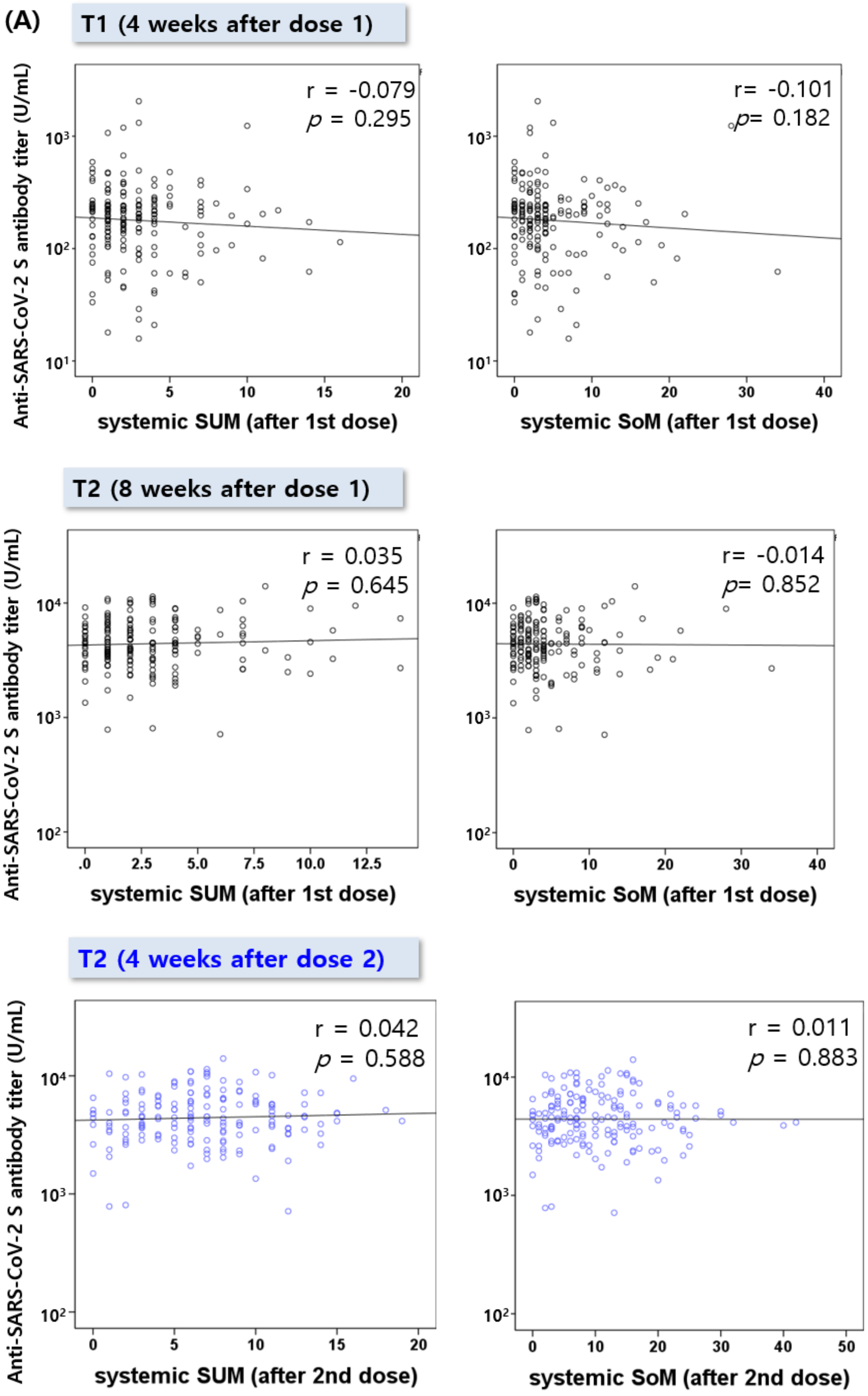

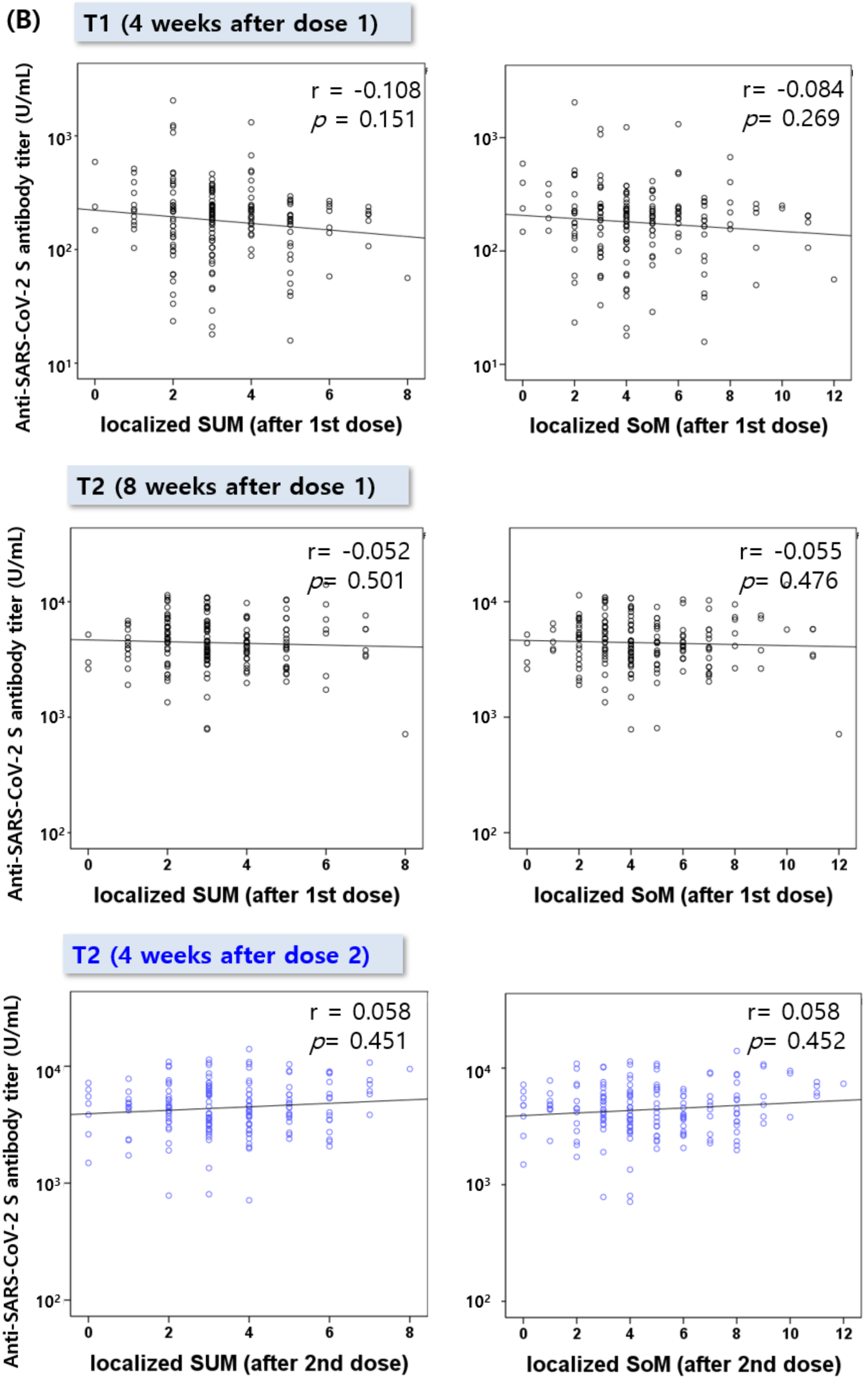
(B)
T1 (4 weeks after dose 1)
r = -0.108
p = 0.151
localized SUM (after 1st dose)
r= -0.084
p= 0.269
localized SoM (after 1st dose)
T2 (8 weeks after dose 1)
r= -0.052
p= 0.501
localized SUM (after 1st dose)
r= -0.055
p= 0.476
localized SoM (after 1st dose)
T2 (4 weeks after dose 2)
r = 0.058
p= 0.451
localized SUM (after 2nd dose)
r= 0.058
p= 0.452
localized SoM (after 2nd dose)
Anti-SARS-CoV-2 S antibody titer (U/mL)

**Supplementary Figure 5.** Relationship of neutralizing antibody response (PRNT $ND_{50}$) with (A) systemic SUM and SOM, and (B) localized SUM and SOM at each time point. PRNT: plaque reduction neutralization test.

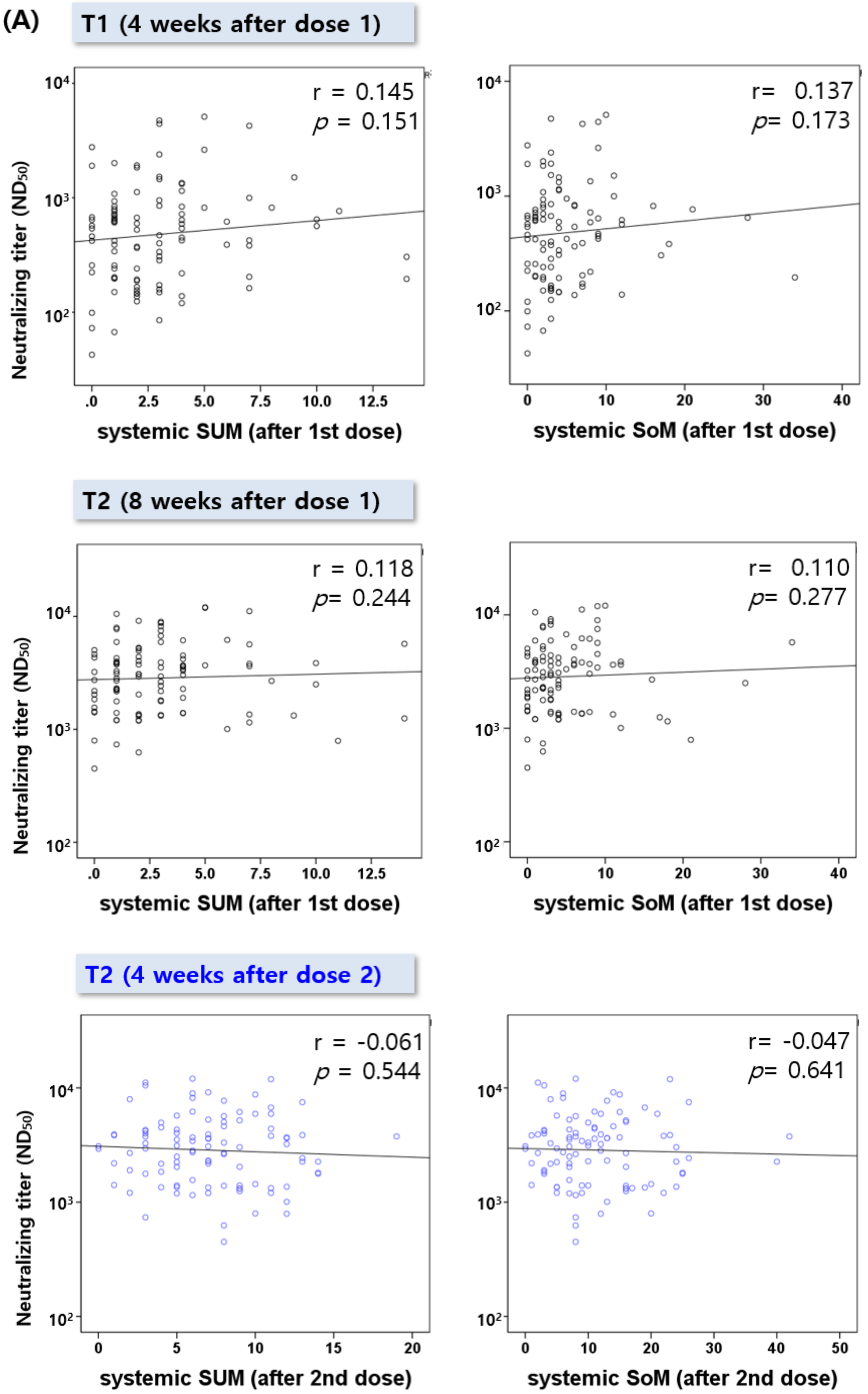

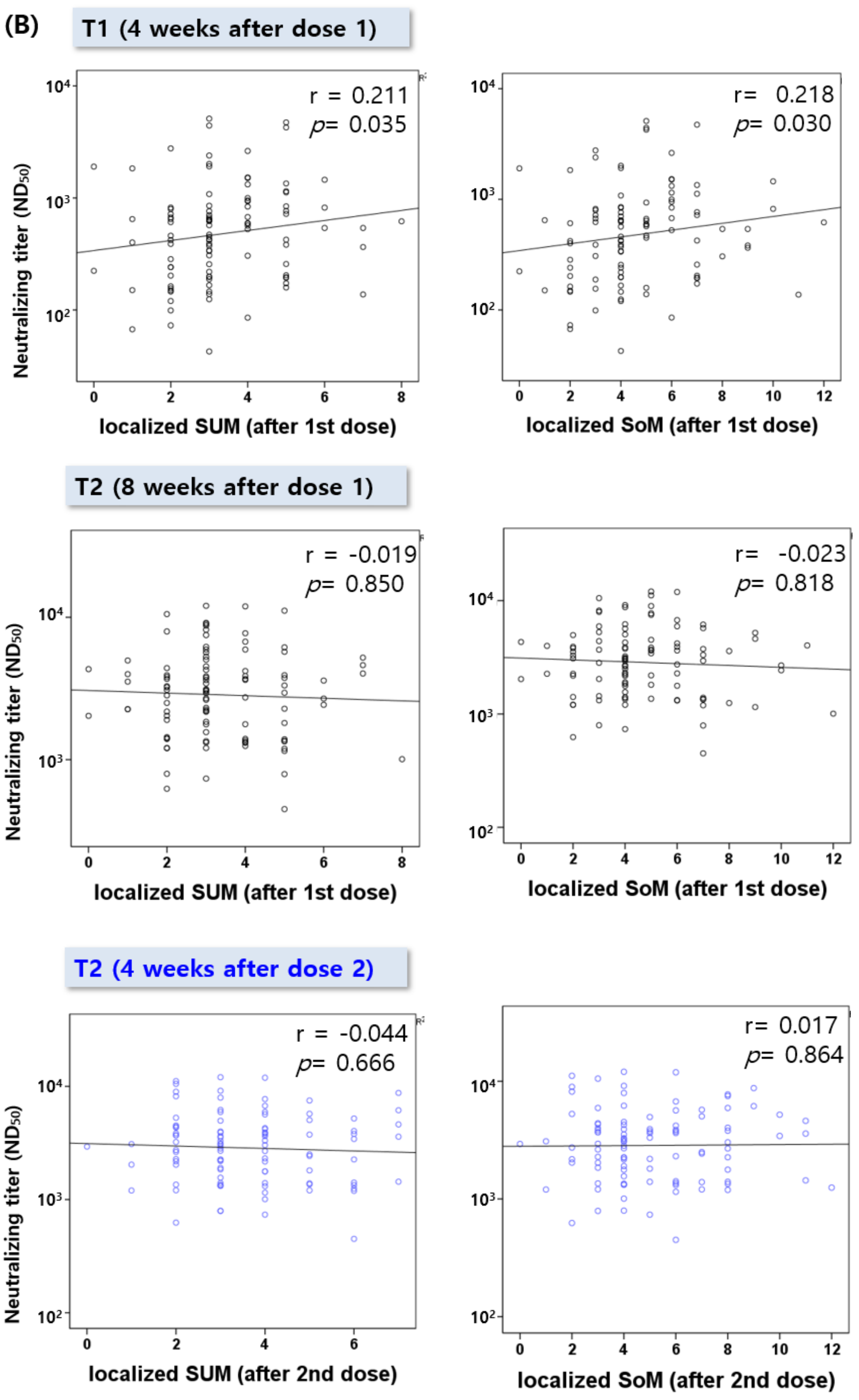
(B)
T1 (4 weeks after dose 1)
r = 0.211
p= 0.035
r= 0.218
p= 0.030
Neutralizing titer (ND50)
localized SUM (after 1st dose)
localized SoM (after 1st dose)
T2 (8 weeks after dose 1)
r = -0.019
p= 0.850
r= -0.023
p= 0.818
Neutralizing titer (ND50)
localized SUM (after 1st dose)
localized SoM (after 1st dose)
T2 (4 weeks after dose 2)
r = -0.044
p= 0.666
r= 0.017
p= 0.864
Neutralizing titer (ND50)
localized SUM (after 2nd dose)
localized SoM (after 2nd dose)
